# Role of diabetes in lung injury from acute exposure to electronic cigarette, heated tobacco product, and combustible cigarette aerosols in an animal model

**DOI:** 10.1371/journal.pone.0255876

**Published:** 2021-08-10

**Authors:** Michella Abi Zeid Daou, Alan Shihadeh, Yasmine Hashem, Hala Bitar, Alaa Kassir, Mohammad El-Harakeh, Nareg Karaoghlanian, Assaad A. Eid, Marwan El-Sabban, Ghazi Zaatari, Ahmad Husari

**Affiliations:** 1 Division of Pulmonary and Critical Care Medicine, Department of Internal Medicine, American University of Beirut, Beirut, Lebanon; 2 Department of Mechanical Engineering, American University of Beirut, Beirut, Lebanon; 3 Center for the Study of Tobacco Products, Department of Psychology, Virginia Commonwealth University, Richmond, Virginia, United States of America; 4 Department of Anatomy, Cell Biology, and Physiological Sciences, Faculty of Medicine, American University of Beirut, Beirut, Lebanon; 5 Department of Pathology & Laboratory Medicine, American University of Beirut, Beirut, Lebanon; University of Western Ontario, CANADA

## Abstract

**Background:**

Patients with diabetes are more vulnerable to the detrimental respiratory effects of combustible cigarette smoke (CS) when compared to the general population. Electronic cigarettes (ECIG) and heated tobacco products (HTP) are marketed as less harmful alternatives to CS. In this study, we compared the effects of acute ECIG, HTP and CS exposure on the lungs of type II diabetes versus non-diabetic mice in an animal model.

**Methods:**

Type II Diabetic (Diab) and Non-Diabetic (Non-Diab) mice were divided into Control, ECIG, HTP and CS groups. Animals were exposed for 6 hrs./day to either air, ECIG, HTP or CS for seven days. Lung injury was determined by a) histopathology, b) wet to dry ratio, c) albumin concentration in bronchoalveolar lavage fluid, d) expression of TNF-α, IL-6, and IL-1 β, e) reactive oxygen species production (ROS), and f) assessment of cellular apoptosis.

**Results:**

Lung histology revealed increased edema and inflammatory cells in diabetic mice exposed to ECIG, HTP and CS. The expression of Inflammatory mediators was, in general, more significant in the Diabetic groups as well. TNF-α expression, for example, was upregulated in Diab + ECIG but not in Non-Diab + ECIG. ROS was significantly increased in Diab + CS, less in Non-Diab + CS and weakly noted in ECIG + Diab. Significant albumin leak was observed in Diab and Non-Diab HTP-exposed animals. CS exposure worsened lung injury in Diab when compared to Non-Diab mice.

**Conclusion:**

Comorbid medical conditions like diabetes may amplify ill effects of CS, ECIG or HTP exposure.

## Introduction

Combustible cigarette smoke (CS) contains toxicants that are harmful to human health and that result in organ damage and early death [1]. Chronic obstructive pulmonary disease (COPD), for example, is associated with CS exposure and was projected to become the third most common cause of death by 2020 [2, 3]. Electronic cigarettes (ECIG) and heated tobacco products (HTP) are marketed as less harmful or not harmful alternatives to combustible cigarettes, and sometimes as effective tools for smoking cessation [4]. ECIGs are battery-powered devices that heat and vaporize a liquid solution that usually contains nicotine, propylene glycol, glycerin, and other ingredients [5]. To attract more users, ECIG manufacturers are now adding a wide range of flavors to the liquid solutions [6]. Some ECIG users are advocating for ECIG exemption from indoor-smoking policies [7]. Despite the inconclusive data on their long-term health effects and population-wide impact on tobacco use patterns, ECIGs continue to penetrate the global markets with increased sales and consumption [8, 9].

Another category of tobacco products is known as heat-not-burn or heated tobacco products (HTP). These products involve electrically heating a tobacco filler similar to that found in combustible cigarettes. HTPs produced by Phillip Morris International are marketed under the IQOS brand, which has been cleared for marketing as a Modified Risk Tobacco Product by the US Food and Drug Administration [10]. IQOS uses a battery-powered heating blade to reportedly heat the filler to a temperature of 350°C, producing an aerosol that mimics that of a combustible cigarette, but with reduced toxicant content [11]. The adverse effects of HTP use are not thoroughly tested and scientifically established.

Type II diabetes is a major global health problem and it is recognized that cardiovascular and respiratory complications are augmented in cigarette smokers [12, 13]. Smoking is associated with significantly higher rates of mortality and morbidity in diabetic than in non-diabetic populations [14]. To the best of our knowledge, the effects of ECIG or HTP on subjects with comorbid medical conditions like diabetes have not been examined. In this study, we examined whether ECIG or HTP exposure exaggerates the respiratory effects in diabetics when compared to non-diabetics in an animal model. In addition, the study compared the effects of ECIG and HTP to the detrimental effects of CS exposure in diabetic and non-diabetic mice.

## Methods

### Animals

All animal procedures were conducted according to the National Institutes of Health (NIH) Guide for the Care and Use of Laboratory Animals and were approved by the Institutional Animal Care and Use Committee at the American University of Beirut. Four-month-old non-obese type II female diabetic FVB-Tg (Ckm-1GF1R*K1003R)1Dlr/J mice (Charles Laboratory, Maine, USA) weighing 22 to 25 grams (Diab) and age matching (Non-Diab) FVB-Tg adult female mice were kept in a temperature-controlled room and on a 12/12 hrs dark/light cycle and were provided standard chow and water access except when mice were placed in the exposure apparatus. Female mice were selected because of their established vulnerability to CS when compared to males. The ECIG, HTP, CS or exposure apparatus (ONARES, CH Technologies, USA) consisted of a smoke generator, mixing/conditioning chamber and a twelve “nose only” rodent exposure carousel. One port was dedicated for sampling analysis, and the remaining 11 ports were used for animal exposure. Non-Diabetic mice were each divided into four groups: Control Non-Diab, Non-Diab + ECIG, Non-Diab + HTP and Non-Diab + CS and each group consisted of 8 animals. Similarly, diabetic mice were divided into: Control Diab, Diab + ECIG, Diab + HTP and Diab + CS with 8 animals in each group as well. Animals were acclimated to retainers for one week prior to placement in the carousel. As described previously [15], mice were then positioned in retainers and placed into the compartments of the carousel and received a continuous flow of either air or air mixed with ECIG or HTP or CS aerosols. Mice were exposed for seven consecutive days, twice daily (am session and pm session). Each session lasted for 3 hrs. Throughout the experiments, animals were evaluated by the research team in the morning and in the afternoon for any signs of distress. The team was trained to look for signs of animal distress that included decreased activity, ruffled hair, hyperventilation, decreased food intake etc.

At the conclusion of the experiment, animals were anesthetized, and the trachea was cannulated with polyethylene tubing. Animals were exsanguinated by severing the aorta and all efforts were made to minimize animal suffering. The diaphragm was dissected to allow free lung expansion. The left lung was clipped, and the right lung was then lavaged three times by slowly instilling 0.5 ml of PBS (Ca^++^ and Mg^++^ free, 37°C) and then gradually aspirating the lavaged fluid. The lower lobe of the left lung was excised for pulmonary water content evaluation. The upper lobe of the left lung was fixed in formalin for pathology examination and TUNEL assay. The remaining right lung lobes were individually frozen in liquid nitrogen for RNA extraction.

### Aerosol generation and exposure protocol

ECIG aerosol was generated using pre-filled V4L CoolCart (3.5 Ohm, 18 mg/mL labeled nicotine concentration) cartomizer cartridges, connected to an automatically activated 4.2 V Vapor Titan Soft Touch battery (Vapor4life, Illinois, USA). To ensure a steady aerosol generation during the 3-hour exposure sessions, cartridges and batteries were replaced every 30 min. As described before, ECIG puff parameters were set at 4 s puff duration, 1.2 L/min flow rate, and 14 s inter-puff interval [16].

HTP aerosols were generated utilizing the ‘I quit smoking system’ (IQOS). The tobacco sticks, holders and the batteries were purchased from the internet vendor (www.IQOS.COM). The holder was fully charged before its use and the tobacco sticks were replaced every 6 min. The puff duration was 2 sec and the time in-between the puffs was 58 sec (i.e. 1 puff for 2 seconds every 1 minute). The puff depth was 35 ml. CS was generated from 3R4F cigarettes (University of Kentucky, Lexington, KY) with 0.9 mg TPM, 9.4 mg tar, and 0.726 mg nicotine per cigarette. The machine was set at one puff every minute with a duration of two seconds per puff and a volume of 35 ml per puff as well. With the current setup, cotinine levels were sampled from different groups. CS animals displayed levels that ranged from (40 to 55 ng/ml) as compared to ECIG and HTP groups where levels were consistently > 100 ng/ml. Cotinine levels signified adequate exposure to HTP and ECIG when compared to CS. With the current utilized protocol, non-diabetic animals would be exposed to levels that will result is measurable lung injury [17]. The differential effects on Diabetic mice can then be ascertained.

### Lung histology

The upper lobe of the right lung was fixed in 10% buffered formalin, embedded in paraffin, sectioned and stained with Hematoxylin and Eosin (H&E). Researchers, blinded to the different animal groups, evaluated the degree of lung injury based on the following histological features: alveolar interstitial wall edema, congestion, degree of inflammatory cell infiltration, and intra-alveolar edema [16].

### Wet-to-dry lung weight

The left lower lobe dissected, weighed and recorded as the wet weight. Samples were then placed in a 95 °C oven to dry for two days. The dried tissue was weighed, and the wet-to-dry ratio (W/D) was then calculated.

### Albumin level

To determine the concentration of albumin in the BALF, an immune-turbidimetric assay was utilized (16). Briefly, agglutination was created by antigen/antibody complex reactions which were then measured turbidimetrically at the clinical chemistry laboratory of the American University of Beirut Medical Center using a Hitachi 912 Autoanalyzer (Roche Diagnostics, Basel, Switzerland).

### Transcription expression profile of inflammatory mediators IL-1β, IL-6, and TNF- α

Quantitative Polymerase Chain Reaction (q-PCR) was used to evaluate inflammatory mediators’ transcriptional levels. RNA was extracted from lung tissues using the TRIzol method (Invitrogen, Carlsbad, CA, USA). We used 1 ml of TRIzol reagent for every 50–100 mg of lung tissue and followed by chloroform extraction. RNA samples were then precipitated and measured using a 260/280 nm absorbance ratio method. Total RNA (5μg) was reverse-transcribed into first strand cDNA. Real time-PCR was performed using the iCycler (Bio-Rad Laboratories, Hercules, CA, USA) with SYBR Green. For every inflammatory mediator measured, a specific primer (Tib-Molbiol, Berlin, Germany) was used to determine its corresponding expression in lung tissues (*IL-1β*: Fw CACCTCTCAAGCAGAGCACAG, Rw GGGTTCCATGGTGAAGTCAAC; *IL-6*: Fw TCCTACCCCAACTTCCAATGCTC, Rw TTGGATGGTCTTGGTCCTTAGCC; *TNF-α*: Fw AATGGGCTCCCTCTCATCAGTTC, Rw TCTGCTTGGTGGTTTGCTACGAC). PCR products and their corresponding melting temperatures were analyzed using the iQ5 Optical System Software" (Bio-Rad Laboratories). Adjustment for loading was accomplished by deducting for local background and standardizing against the cDNA levels of the GAPDH housekeeping gene (*GAPDH*: Fw GTATTGGGCGCCTGGTCACC, Rw CGCTCCTGGAAGATGGTGATGG).

### Assessment of reactive oxygen species (ROS) production

Dihydroethidium (DHE) (Invitrogen, Molecular Probes, USA) at a concentration of 10 μmol/L, dissolved in DMSO, was applied to lung sections and then incubated in a light-protected humidified chamber at 37 °C for 15min. The obtained fluorescent images were then scanned for a signal with a confocal microscope (LSM 710, Zeiss, Germany). As described before, Ethidium bromide was excited at 488 nm, and fluorescence was detected at 560 nm long-pass filter. Mean fluorescence intensity of the digitalized image was measured with Image software [National Institutes of Health (NIH), Bethesda, MD] [16].

### Assessment of apoptosis—TUNEL assay

The terminal deoxynucleotidyl transferase-mediated dUTP nick-end labeling (TUNEL) assay was used to determine the level of DNA fragmentation as a measure of apoptosis. Fluorescein-conjugated dUTP incorporated in nucleotide polymers was detected and analyzed using fluorescence microscopy (Zeiss LSM 710, Germany). Positive and negative controls were used to verify the specificity of the TUNEL assay. TUNEL-positive nuclei were distinguished from the TUNEL-negative nuclei by counterstaining with Hoechst 33258.

### Statistical analysis

Results were expressed as the mean ± standard error of the mean (SEM). Statistical comparisons were performed using one-way analysis of variance (ANOVA), followed by Tukey’s multiple comparisons test. The P value was determined and considered significant for P < 0.05.

## Results

One animal in the Diab + CS expired on day six of CS exposure. All other animals in the different groups (Diab and Non-Diab) survived the experiment. CS animals displayed limited decrease in body weight that was statistically non-significant.

### Lung histology

**Fig 1:**
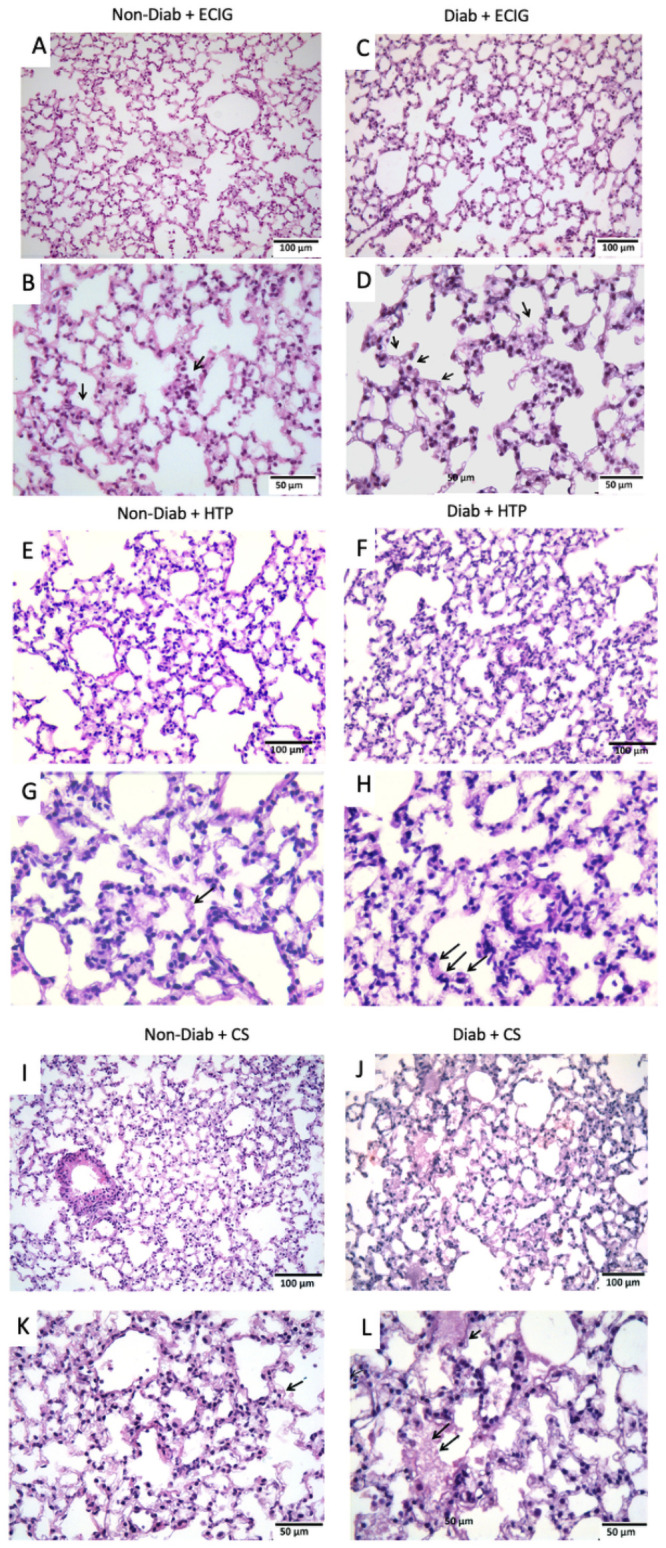
Histology of lungs of Non-Diabetic vs Diabetic after seven days of ECIG, HTP and CS exposure. Non-Diabetic lungs + ECIG showing limited lung injury with thin interstitial alveolar wall and fine capillary vessels (Fig 1A; magnification x 20). Rare inflammatory cells in the wall and intra-alveolar spaces are noted (Fig 1B; magnification x 40). Sections from Diabetic + ECIG lungs demonstrate limited areas of thickening of the interstitial wall, capillary congestion and limited areas of inflammation (Fig 1C; magnification x 20). Arrows point to a significant presence of inflammatory cells (Fig 1D; magnification x 40). Non-Diabetic lungs + HTP showing patches of alveolar wall edema and capillary congestion (Fig 1E; magnification x 20). Those findings were significantly worse in Diabetic lungs exposed to HTP (Fig 1F; magnification x 20). Arrows point to presence of inflammatory cells (Fig 1H; magnification x 40). Non-diabetic lungs showing significant lung injury with thick interstitial alveolar walls and capillary congestion (Fig 1J; magnification X20). inflammatory cells in the wall and intra-alveolar spaces are noted (Fig 1K; magnification x40). Sections from Diabetic lungs demonstrate worsening lung injury when compared to non-diabetic animals with destruction of the alveolar architecture and edematous distension of the alveolar sacs (Fig 1L; magnification x 20). Worsening recruitment of inflammatory cells is now observed as well (Fig 1M; magnification x 40). Non-Diab + HTP lungs revealed patches of alveolar wall edema and capillary congestion (Fig 1E and 1F). These findings were significantly worse in Diab + HTP when compared to Non-Diab +HTP. Non-Diab + CS and Diab + CS lungs demonstrated significant acute lung injury in both groups. Diab + CS lung injury was significantly worse when compared to Non-Diab + CS with worsening edematous and thickened alveolar walls, in addition to significant infiltration by inflammatory cells (Fig 1k and 1L).

H&E staining of lung sections from Non-Diab + ECIG essentially displayed “normal” alveolar structure with isolated foci of infiltration of inflammatory cells (Fig 1A and 1B). Diab + ECIG demonstrated worsening infiltration of inflammatory cells (macrophages and lymphocytes) around the bronchioles and into the lung parenchyma. Edematous and thickened alveolar walls were also noted (Fig 1C and 1D).

### W/D ratio and albumin leak

**Fig 2:**
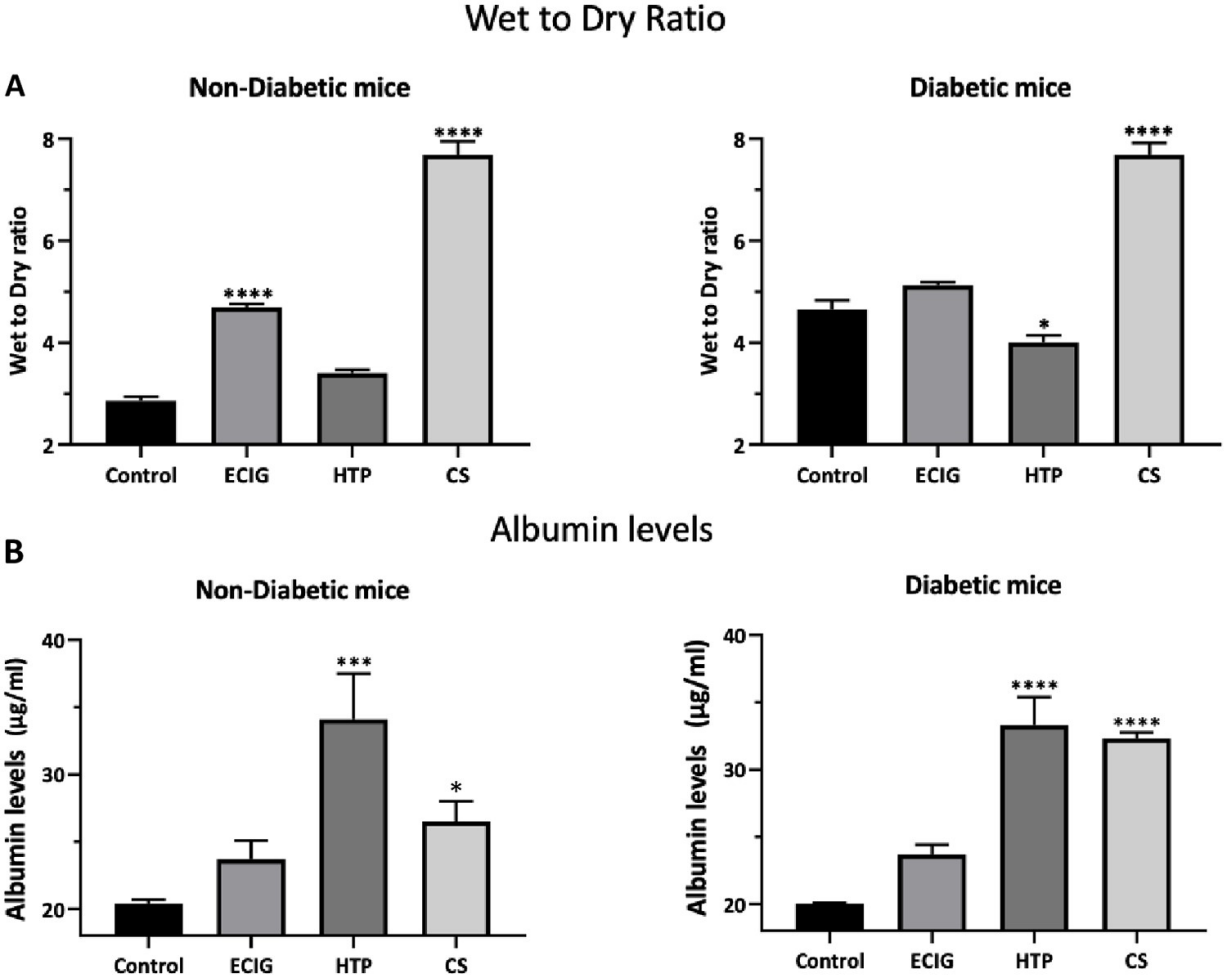
Mean wet to dry ratio (W/D) (A)and albumin level in the BALF (B) of Control, ECIG and CS for Non-Diabetic and Diabetic mice. Error bars represent SE. Asterisks indicate statistically significant associations (*P-value: < 0.05, **P-value: < 0.005, **** P-value: < 0.0001).

In the diabetic group, there was a significant increase in the W/D ratio, when compared to Control, for animals exposed to CS. ECIG demonstrated no significant increase in W/D ratio when compared to Control in Diabetic mice, however, a significant increase was detected in ECIG-exposed group compared to Control in Non-Diab group. As for albumin leak into the BALF, no significant increase in albumin leak was noted in both ECIG animals (Non-Diab and Diab), but, HTP and CS animals in both groups (Non-Diab and Diab) demonstrated significant albumin leak into the BALF when compared to their respective Control (Fig 2A and 2B).

### Inflammatory mediators

#### TNF-α

**Fig 3:**
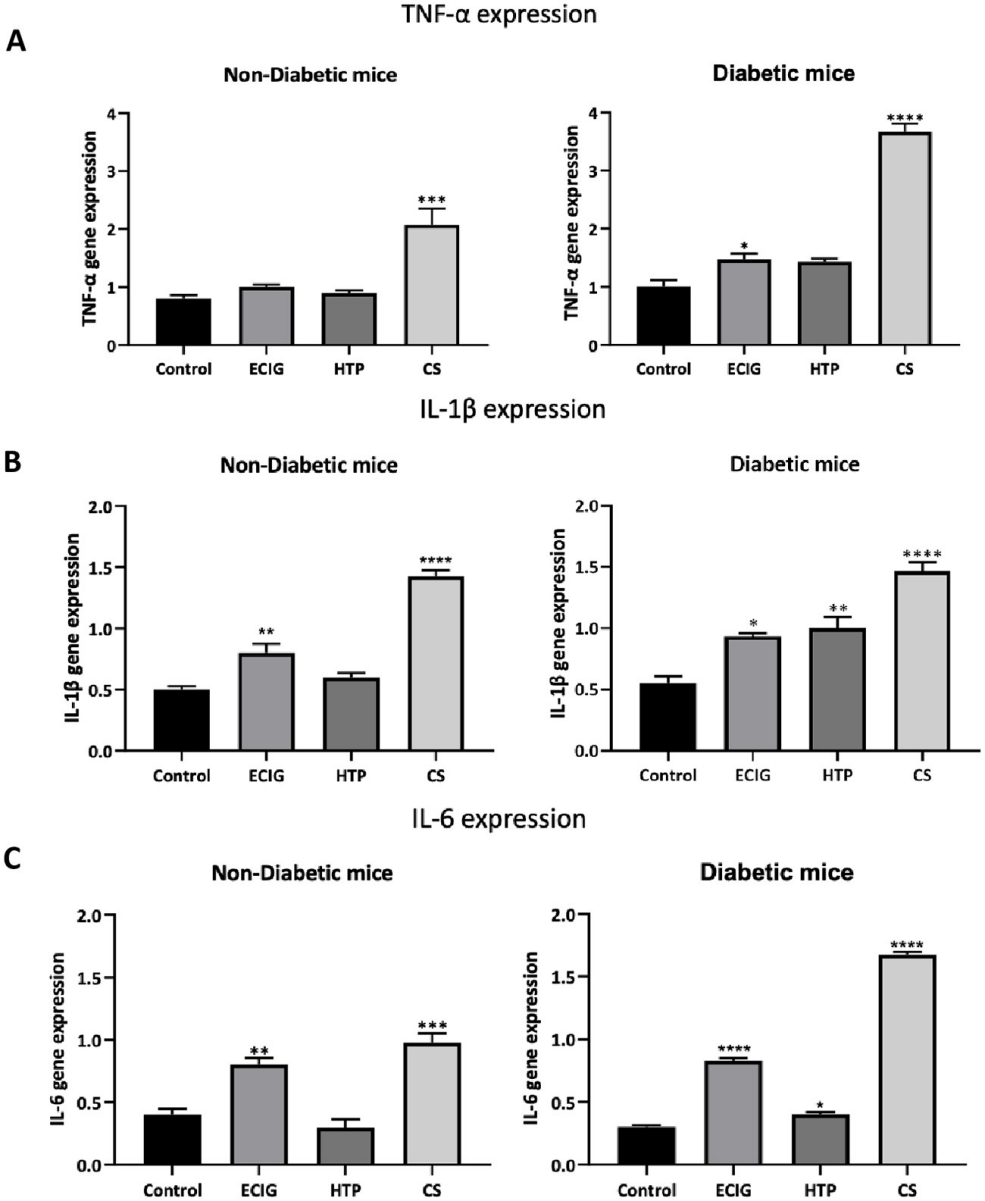
Transcriptional expression of TNF-α (A) IL-1β (B) and IL-6 (C) of Control, ECIG and CS for Non-Diabetic and Diabetic mice. Expression of inflammatory mediators was assessed by real-time PCR. Error bars represent SE and sterisks indicate statistically significant associations (* = P-value: < 0.05, ** = P-value: < 0.005, *** = P-value: < 0.0005, **** = P-value: < 0.0001).

Exposure to ECIG or HTP did not result in an increase in TNF-α in the Non-Diabetic group. On the other hand, Diab + ECIG demonstrated a significant increase in TNF-α expression when compared to Non-Diab Control (Fig 3A). CS exposure resulted in a significant increase TNF-α in expression in Non-Diabetic and diabetic groups as well when compared to Non-Diab and Diab groups respectively.

#### IL-1β

Exposure to ECIG resulted in increased IL-1β expression in both Non-Diab and Diab groups when compared to Control. Exposure to HTP, however, resulted in a significant increased IL-1β expression only in the diabetic group (Fig 3B). Non-Diab + HTP IL-1β expression was similar to Control. CS exposure was associated with a surge IL-1β expression in both groups (Diab and non-Diab). The intensity of IL-1β expression of CS groups was significantly higher when compared to ECIG and HTP for Diab and Non-Diab groups respectively.

#### IL-6

The intensity of IL-6 expression was significantly increased in both Non-Diab + ECIG and Diab + ECIG when compared to Control (Fig 3C). HTP exposure did not result in any significant increase in the IL-6 expression for Non-Diabetic group, however a significant increase of IL-6 expression in Diab mice exposed to ECIG when compared to Control Diab. As described previously, CS exposure, IL-6 expression was significantly increased in both groups when compared to Control.

### Oxidative stress and apoptosis

**Fig 4:**
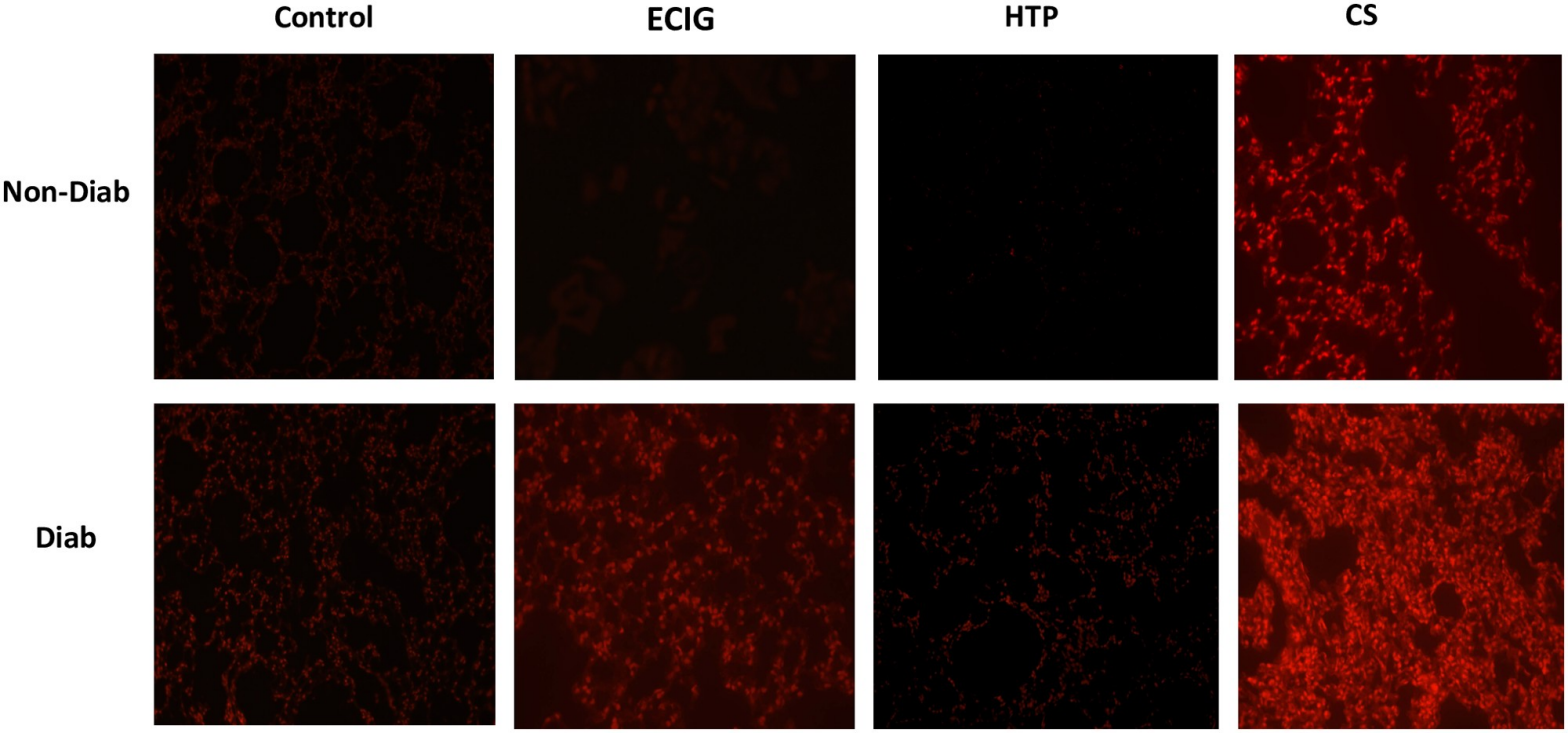
ROS detection in lung tissues. Five μm thickness slides of lung sections for Control, ECIG and CS from Non-Diabetic and diabetic mice were incubated with Dihydroethidium (DHE), and fluorescent images of ethidium-stained tissue were analyzed. ROS levels were significantly higher in ECIG and CS and exposed diabetic mice when compared to ECIG and CS Non-Diabetic mice exposure respectively.

HTP exposure did not result in any significant OS in Diab and Non-Diab animals. Diabetic mice exhibited a significant increase in ROS production in ECIG and CS groups when compared to Control and Non-Diabetic groups. CS-induced ROS were significantly higher in Diab + CS when compared to Non-Diab + CS. In the Non-Diab mice groups, OS was only observed in CS + Non-Diab when compared to Control (Fig 4).

**Fig 5:**
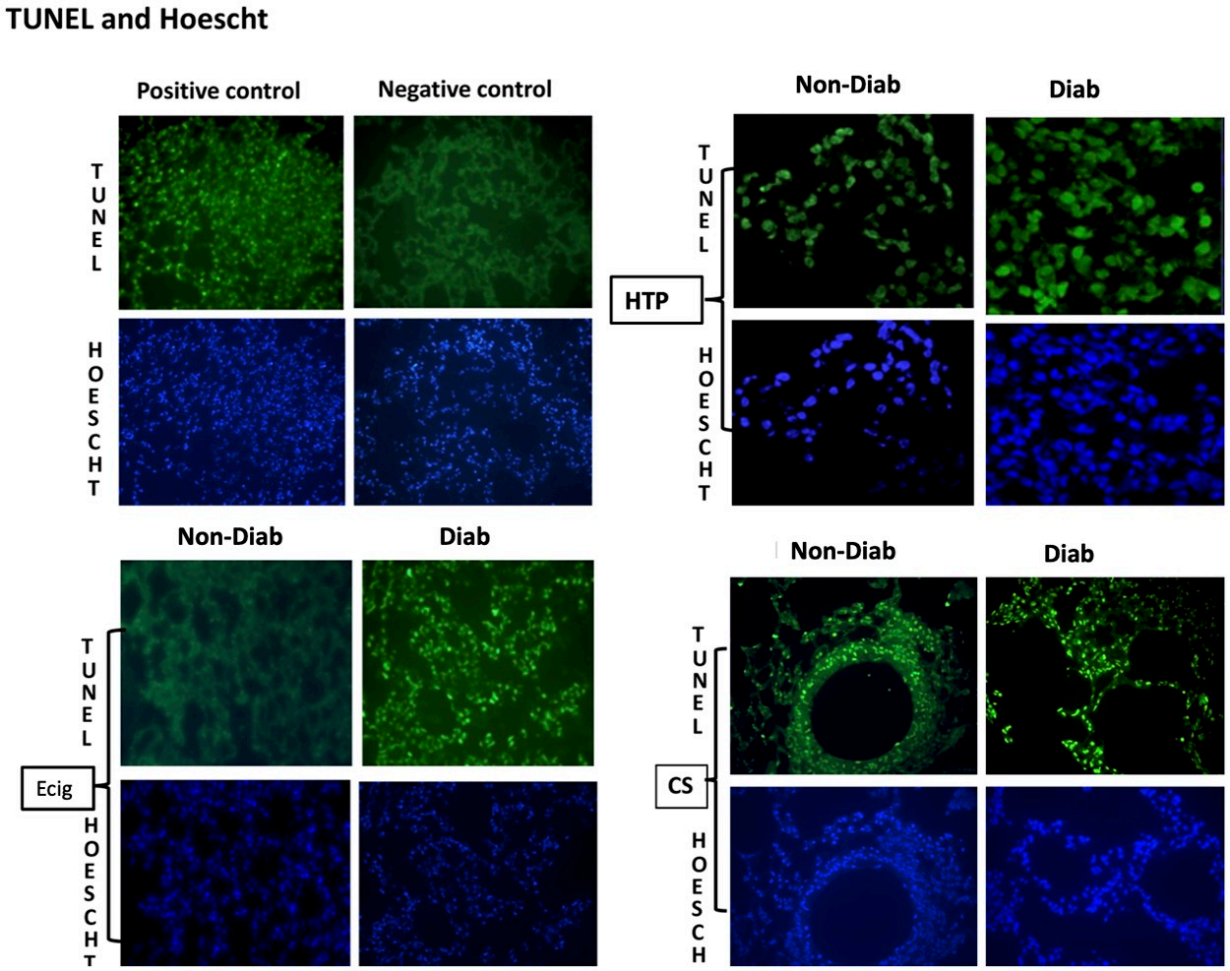
Cell death assessment. Terminal deoxynucleotidyltransferase-mediated dUTP nick-end labeling (TUNEL) and Hoechst staining of lung sections for Control, ECIG and CS from Non-Diabetic and diabetic mice. Significant increase in apoptotic activity was noted in ECIG and CS and exposed diabetic mice when compared to ECIG and CS Non-Diabetic mice respectively.

Similarly, a significant increase in the number of TUNEL positive, apoptotic nuclei, indicating cellular death, was detected in Diab + ECIG + and Diab + CS mice when compared to Control. The number of TUNEL-positive apoptotic nuclei was significantly higher with CS compared to ECIG exposure (Fig 5). HTP exposure did not result in any significant increase in apoptosis in Diab + HTP nor Non-Diab +HTP.

## Discussion

This study was designed to examine how diabetes mediates lung injury on exposure to ECIG, HTP, or CS aerosols. We found that when exposed to ECIG aerosols the lungs of diabetic mice demonstrated a significant increase in albumin leak, a surge in the expression of IL-1β, IL-6 and TNF-α and worsening OS and apoptosis. In addition, histopathological assessment of the lung tissue demonstrated worsening alveolar edema and infiltration by inflammatory cells. Non-Diabetic mice, exposed to ECIG aerosols, demonstrated lower severity of lung injury with ECIG exposure. IL-1β expression was the only tested inflammatory marker that was increased with ECIG exposure, but no significant surge was observed in IL-6, ROS production or apoptosis when compared to Control.

As for HTP exposure, there was no detectable increase in inflammation or lung injury in the Non-Diabetic group. Diab + HTP, however, was associated with a significant increase in TNF-α, IL-1β and when compared to Control. HTP exposure was, however, associated with significant albumin leak into the BALF of diabetic and nondiabetic mice. Similar observations were recently described by Bhat et al where significant albumin leak was also observed in animals exposed to HTP [18]. What triggers albumin leak after exposure to HTP will warrant future studies and assessment. When HTP groups (Diab and not Diab) were compared to ECIG and CS groups, we concluded that HTP exposure was associated with the lowest injury to the lungs in this animal model. As for CS exposure, there was an increase in ROS, apoptosis and inflammatory mediators’ expression that was more pronounced in the diabetic group when compared to the Non-Diabetic group with similar CS exposure. The above findings suggest that diabetic mice are more vulnerable when exposed to external irritants such as those present in and ECIG, HTP or CS aerosol.

The association between comorbid health conditions and their augmentation of lung injury and inflammatory response secondary to illnesses and infections was reported in human and animal studies [19, 20]. Diabetes, in particular, is known to exacerbate systemic inflammation as evidenced by “priming the lung” with higher serum h*igh mobility group box 1* (HMGB1), higher cytokine levels and enhanced lung injury in a systemic inflammation animal model [21, 22]. In addition, diabetes augments oxidant and pro-inflammatory cytokines expression in lung, liver, and heart in a septic animal model [23]. The negative impact of diabetes on the lungs is observed in human epidemiological studies as higher fasting insulin and insulin resistance is associated with worsening pulmonary function testing [24]. Other comorbid medical conditions have demonstrated an exacerbated response to CS with worsening lung injury as well. Crothers et al. [25], for example, examined the interplay of human immunodeficiency virus (HIV) as a comorbid medical condition with CS exposure and lung injury. The study observed worsening vulnerability of HIV-positive veterans to CS exposure by showing increased respiratory symptoms, COPD, and bacterial pneumonia which all resulted in their increased mortality and decreased quality of life.

## Conclusion

This study highlights the importance of comorbid health conditions in amplifying inhalational lung injury in ECIG, HTP and combustible tobacco products consumers. While limited or no findings were noted in the Non-Diab mice exposed to HTP or ECIG for one week of exposure, significant lung injury was noted with ECIG exposure and to a lesser extent with HTP resulting in alveolar injury, increased OS and apoptosis and a significant surge in some of the inflammatory markers in diabetic mice.

A Limitation of our study include the acute duration of animal exposure as our study did not explore the chronic effects of prolonged exposure. It is possible that chronic studies may reveal reactive animal adaptation and adjustments resulting in different observations. Another limitation of this study is the assigned amount of daily exposure to ECIG, HTP and CS and the one time point of seven days after which the experiment was concluded. More studies with prolonged exposure, different exposure regimens and multiple time points need to follow this study.

As more novel tobacco products are introduced into the global markets, it is critical to assess their harm and health effects not only on healthy subjects but also to extend the assessment to subjects with additional comorbid health conditions, such as diabetes.

## Abbreviations

COPD: Chronic obstructive pulmonary disease
CS: Cigarette smoke
ECIG: Electronic cigarettes
HTTP: Heated tobacco products
OS: Oxidative stress
ROS: Reactive Oxygen Species
TUNEL: Terminal deoxynucleotidyl transferase-mediated dUTP nick-end labeling
